# Automated cardiac arrest detection using wrist-derived photoplethysmography during withdrawal of life-sustaining treatment: a prospective clinical validation study

**DOI:** 10.1016/j.lanepe.2026.101791

**Published:** 2026-07-24

**Authors:** Roos Edgar, Catharina E. Jansen, Lente R. Pol, Kambiz Ebrahimkheil, Ruud C. van Kaam, Eelko Ronner, Marc A. Brouwer, Rypko J. Beukema, Aysun Cetinyurek-Yavuz, Peter C. Stas, Eric Boersma, Cornelia W.E. Hoedemaekers, Niels van Royen, Judith L. Bonnes

**Affiliations:** a Department of Cardiology, Radboud University Medical Center, Geert Grooteplein Zuid 10, 6525 GA, Nijmegen, the Netherlands; b Corsano Health, Wilhelmina van Pruisenweg 35, 2595 AN, The Hague, the Netherlands; c Intensive Care, Radboud University Medical Center, Geert Grooteplein Zuid 10, 6525 GA, Nijmegen, the Netherlands; d Department of Cardiology, Reinier de Graaf Hospital, Reinier de Graafweg 5, 2625 AD, Delft, the Netherlands; e Radboud Institute for Health Sciences, Health Evidence, Section Biostatistics, Nijmegen, the Netherlands; f Department of Cardiology, Cardiovascular Institute, Erasmus MC, University Medical Center, Dr. Molewaterplein 40, 3015 GD, Rotterdam, the Netherlands

**Keywords:** Out-of-hospital cardiac arrest, Automated detection, Wearable, Photoplethysmography, Non-shockable cardiac arrest

## Abstract

**Background:**

Automated cardiac arrest detection and alerting using wearable technology has the potential to shorten recognition delays for unwitnessed out-of-hospital cardiac arrest. In DETECT-1A and -1B, a photoplethysmography-based detection model was developed and validated in patients with induced cardiac arrest. This study evaluates model performance in true cardiac arrests following withdrawal of life-sustaining treatment.

**Methods:**

Prospective, single-center study in adult ICU patients with planned withdrawal of life-sustaining treatment. Patients wore a photoplethysmography-wristband (CardioWatch) until death. Continuous ECG and invasive arterial pressure served as reference standards. The previously developed rule-based algorithm was refined using two training cohorts and evaluated in a separate test cohort. Endpoints were sensitivity for cardiac arrest detection and false positive alerts.

**Findings:**

Forty-four patients were included (training 1: n = 10; training 2: n = 11; test: n = 23), median age 65 years; 75% male, all with non-shockable cardiac arrest. Sensitivity for cardiac arrest detection was 100% (10/10; 95% confidence interval [CI] 66–100%) and 90% (9/10; 95% CI 54–99%), in training 1 and 2, respectively. In the test set, sensitivity was 100% (23/23; 95% CI 82–100%), with one false positive alert. Cardiac arrest was detected at a mean arterial pressure of 30 mmHg (IQR 24–35) and pulse pressure of 13 mmHg (IQR 11–19).

**Interpretation:**

Cardiac arrest can be detected with high sensitivity using wrist-derived photoplethysmography, providing first evidence on model performance in true cardiac arrest, specifically in non-shockable cases. Findings support further development of wearable-based cardiac arrest detection technologies to enable earlier recognition for unwitnessed cardiac arrest.

**Funding:**

Dutch Heart Foundation, Radboudumc.

**Research in context**

**Evidence before this study**

We searched PubMed for research published in English up till 13 February 2026, with search terms expressing cardiac arrest (e.g. “cardiac arrest”, “out-of-hospital cardiac arrest”, “OHCA”, “sudden cardiac death”, “resuscitation”) and monitoring technologies (“wearable,” “device,” “sensor,” “smartwatch”, “continuous monitoring”, “automated detection”). Several initiatives have recently focused on developing wearable-based cardiac arrest detection technologies, predominantly using photoplethysmography as the main sensor. Existing studies include mostly feasibility studies, and some development and validation reports, including one commercially available device. The existing studies that use PPG-models for cardiac arrest detection fall into two categories: those using simulated circulatory arrest in healthy volunteers, and those using induced cardiac arrest in patients during cardiac procedures. However, validation in true, non-induced cardiac arrest remained lacking in all devices, and the European Resuscitation Council Guidelines 2025 recommend implementation of wearable cardiac arrest detection only within formal research programs.

**Added value of this study**

The DETECT-program is set up to develop a wristband-based technological solution for automated cardiac arrest detection and alarming in a home setting. Following algorithm development and validation in induced circulatory arrest, DETECT-1C represents the next step toward real-world applicability. This prospective study evaluates the photoplethysmography-based algorithm in patients experiencing true cardiac arrest following withdrawal of life-sustaining treatment. Relying on wrist-derived photoplethysmography with continuous invasive blood pressure and ECG as reference standards, cardiac arrest could be detected with high sensitivity and a low number of false positive alerts in an independent test cohort.

**Implications of all the available evidence**

Wearable biosensor technologies may offer a solution for automated cardiac arrest detection and alarming in a home setting. DETECT-1C is the first study to validate a cardiac arrest detection algorithm in true, non-induced cardiac arrest and specifically in non-shockable rhythms, which is the arrest type most prevalent in the community yet previously unaddressed. These findings are an important step toward real-world applicability across the full spectrum of cardiac arrest presentations. This potentially lifesaving technology warrants further validation, aiming to enable earlier recognition of out-of-hospital cardiac arrest.

## Introduction

Out-of-hospital cardiac arrest is a leading cause of death with an incidence of 50–100 cases per 100,000 person-years annually.^1^ When cardiac arrest is witnessed and cardiopulmonary resuscitation (CPR) is initiated promptly, survival can reach up to 30–50%.^2^^,^^3^ In contrast, in the roughly half of cases that occur without a witness, survival is poor (<5%) because recognition is delayed and the emergency response starts too late.^4^^,^^5^ Wearable-based automated cardiac arrest detection and activation of the chain of survival has been proposed to shorten recognition delays.^6^^,^^7^ Several initiatives have recently focused on developing such technologies.^8^, ^9^, ^10^, ^11^, ^12^, ^13^

Most cardiac arrest detection models under investigation use photoplethysmography as the main sensor technology, offering a direct representation of the blood circulation by non-invasively measuring changes in blood volume in the microvascular bed.^14^^,^^15^ The first smartwatch with loss-of-pulse functionality is now commercially available; however, its algorithm was developed and tested exclusively in simulated circulatory arrests in healthy volunteers/stunt persons and sensitivity was modest (53–72%).^13^ Other feasibility studies have used patient data, primarily from induced shockable arrests.^11^^,^^12^ In the DETECT-1A and -1B studies, a wrist-derived photoplethysmography-based cardiac arrest detection model has been developed and was externally validated using data from patients with induced, short-lasting cardiac arrest.^16^^,^^17^

Despite promising first results, all existing models lack validation in patients with true cardiac arrest including non-shockable heart rhythms,^18^ i.e. asystole and pulseless electrical activity. Withdrawal of life-sustaining treatment due to a poor prognosis offers a unique opportunity to evaluate cardiac arrest detection models in a controlled clinical setting, and also specifically in non-shockable cardiac arrests.^19^ In this DETECT-1C study, we evaluated and tested the performance of the previously developed photoplethysmography-model in detecting true cardiac arrest in patients admitted to the intensive care unit (ICU) in whom life-sustaining treatment was withdrawn.

## Methods

### Study design and participants

DETECT-1C was a prospective, single-center study in adult patients admitted to the ICU in whom life-sustaining treatment was planned to be withdrawn due to a poor prognosis. Assessment of prognosis and the decision to withdraw life sustaining treatment was made exclusively by treating clinicians, not involving the research team. Exclusion criteria were absence of an arterial line, baseline mean arterial pressure (MAP) of <60 mmHg despite administration of vasopressors, known bilateral hemodynamically relevant subclavian artery stenosis, and medical issues that interfere with wearing of the CardioWatch wristband. Patients who were scheduled for a heart-beating donor procedure were also not eligible for inclusion.

### Data collection

Prior to withdrawal of life-sustaining treatment, a photoplethysmography-wristband (CardioWatch 287-2, Corsano Health, The Hague, The Netherlands) was attached to the left or right wrist of the patient, depending on the presence of venous/arterial lines and the preference of relatives. The wristband was removed after death was confirmed by the treating physician. Photoplethysmography data (multi-wavelength [green, red, infrared]; sample frequency 32 or 128 Hz) were recorded and sent by a Bluetooth-connected smartphone (Samsung Galaxy A40, Android OS, Samsung Electronics) to a protected cloud. Continuous invasive blood pressure and electrocardiography (ECG) data were collected as reference standard, using the ICM + software (University of Cambridge, United Kingdom). Collected baseline parameters included age, sex (as recorded in the electronic patient files), medical history, underlying etiology, skin type (Fitzpatrick scale), and arm hair density.^20^^,^^21^ The data were fully anonymized.

### Cardiac arrest detection algorithm

We applied the photoplethysmography-algorithm for cardiac arrest detection that was developed in DETECT-1A and externally validated in patients with induced shockable cardiac arrest in DETECT-1B.^16^^,^^17^ Cardiac arrest detection was assessed during post-processing using all recorded photoplethysmography data. The algorithm used the averaged photoplethysmography signals from two green photoplethysmography channels. The photoplethysmography signals were preprocessed with a second-order Butterworth bandpass filter to reduce noise, deploying a 0.5–4 Hz bandwidth, and down sampled to 32 Hz. Then, the filtered photoplethysmography signals were run through the algorithm for cardiac arrest detection. In this algorithm, the baseline photoplethysmography amplitude is determined for each patient based on the previous 2 h of photoplethysmography recordings. If the photoplethysmography amplitude decreases with 67%, there is a possibility of a cardiac arrest. Next, the signal quality index is determined. This signal quality index comprises morphological features that determine whether the remaining photoplethysmography activity corresponds to photoplethysmography pulsations. An alert is triggered in case of a low amplitude and a poor signal quality index for 10 s or longer, indicating that no photoplethysmography pulsations are detected. If a return of four photoplethysmography pulsations is detected (i.e. in case of a false alarm), the cardiac arrest alarm is terminated. After termination, a new alert can subsequently be triggered if the detection criteria are met, allowing multiple alerts per patient. A detailed description of the workflow of the photoplethysmography-algorithm can be found in the DETECT-1A paper.^16^ Data analyses were performed in Python (version 3.11.9, Anaconda Software Distribution, Anaconda Inc.; 2021).

### Cardiac arrest definition

The time of cardiac arrest was defined as the absence of pulse in the arterial blood pressure signal, defined as pulse pressure <5 mmHg.^19^^,^^22^ The underlying cardiac arrest rhythm was determined at this time point. Death was confirmed by the treating physician and was not determined using wristband data. The following timestamps were recorded; 1) the time of withdrawal of life-sustaining treatment, 2) the time of cardiac arrest, and 3) the time of confirmation of death by the treating physician.

### Cardiac arrest recognition by the algorithm and algorithm refinement

All ECG and blood pressure data were reviewed for cardiac arrest events by two investigators (RE, CJ). Cardiac arrest alerts generated by the photoplethysmography-based cardiac arrest detection algorithm were assessed during post-processing. The algorithm developer (KE) running the cardiac arrest detection algorithm had no access to the ECG and blood pressure data. Event adjudication was blinded.

Alerts were classified into three categories. First, an alert was classified as true positive if it occurred after withdrawal of life-sustaining treatment within a time window from 15 min before to 2 min after the time point at which the pulse pressure fell below 5 mmHg. This definition was chosen because hemodynamic decline after withdrawal of life-sustaining treatment is gradual, with progressive decreases in blood pressure, heart rate, and oxygen saturation. Second, alerts occurring earlier were classified as clinically relevant if the alert was accompanied by hemodynamic instability, defined as MAP ≤45 mmHg or pulse pressure ≤15 mmHg,^23^, ^24^, ^25^ and if the alert was cancelled after recovery of blood pressure. This definition and threshold were chosen because pressures below this level are considered insufficient to ensure adequate cerebral perfusion.^25^ Third, all remaining alerts for which reference data were available were classified as false positives. Alerts without an available reference standard were excluded from classification, except for the final alert before death. This alert was evaluated against the documented time of death.

As this was the first formal evaluation of a cardiac arrest detection algorithm in non-induced cardiac arrest, the rule-based photoplethysmography-algorithm was iteratively optimized in two cohorts of 10 patients. Within each cohort, sensitivity for cardiac arrest detection and false positive alerts were assessed. Based on the results, algorithm parameters were adjusted and the performance was re-evaluated within the same cohort. Afterwards, the final algorithm was evaluated in an independent test cohort of at least 20 patients with both photoplethysmography signals and reference measurements available. Assuming an expected sensitivity of 95%, 20 cardiac arrest events yield an approximate margin of error of 11%.

### Endpoints

The primary endpoint was the sensitivity for the detection of cardiac arrest. Secondary endpoints included false positive cardiac arrest alarms, the positive predictive value for cardiac arrest detection, and the MAP and pulse pressure at the algorithm's time of cardiac arrest detection.

### Statistical analysis

Baseline characteristics were presented as means ± standard deviations or medians with interquartile ranges (IQR), and categorical variables as counts with percentages. Algorithm performance was evaluated using sensitivity (recall), number of false positive cardiac arrest alerts, and the positive predictive value (PPV, precision). In addition, specificity, F1-score, and accuracy were reported and can be found in Supplement 2. Sensitivity/recall was defined as the number of correctly identified cardiac arrest events divided by the total number of cardiac arrests, assessed at event level. To prevent overestimation of performance in a cohort with 100% event rate, alerts were only classified as true positive if they occurred within a specific time window as described above. PPV/precision was defined as the number of correctly identified cardiac arrests divided by the sum of correctly identified cardiac arrests and false positives. Sensitivity and PPV were expressed as percentages, whereas recall and precision were expressed as proportions. All metrics were reported with 95% confidence intervals (CI), calculated using the Wilson score method with continuity correction. The MAP and pulse pressure at the time of cardiac arrest detection were reported as medians with IQR. The MAP was calculated as the time-averaged arterial pressure by integrating the continuous arterial pressure waveform. Statistical analyses were performed in SPSS (IBM Corp. Released 2020. IBM SPSS Statistics for Windows, version 29. Armonk, NY: IBM Corp).

### Ethics review

The study protocol was reviewed by the Medical Ethics Committee East-Netherlands and the local ethics committee of Radboud University Medical Center, which determined that it was not subject to the Dutch Medical Research Involving Human Subjects act (WMO). Therefore, a waiver was granted (date 9 October 2023; reference number 2023-16743). Informed consent was obtained verbally from the patient or their relative(s), considering the sensitive care setting. Details regarding the informed consent procedure are described in Supplement 1.

### Role of the Funding source

This research project is financed by public-private partnerships allowance made available by Top Sector Life Science & Health to Hartstichting/Radboudumc to stimulate public-private partnerships (grant numbers 2021B006, R0007420). The funder of the study had no role in study design, data collection, data analysis, data interpretation, or writing of the report.

## Results

### Baseline characteristics

**Fig. 1:**
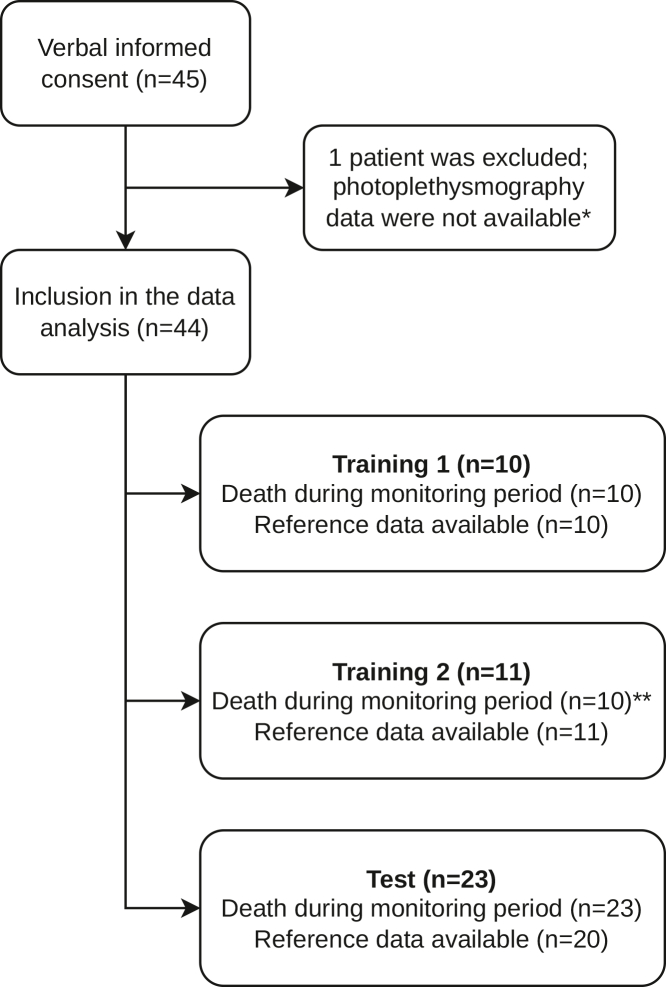
**Study flowchart.** ∗One patient was excluded because of unavailability of photoplethysmography data, caused by a technical issue of the wristband. ∗∗In Training 2, one study participant was discharged from the ICU before circulatory arrest because of a prolonged time to death, and the wristband was detached upon leaving the ICU. The collected photoplethysmography data during the ICU stay were used to assess false positive alerts. Unavailability of reference data in the test set was related to technical issues/human failure.

A total of 45 patients were included between February 12, 2024 and October 27, 2025. One patient was excluded because of unavailability of photoplethysmography data due to device failure. Thus, 44 patients were included in the data analysis, see study flowchart in Fig. 1.

The study participants had a median age of 65 years (IQR 57–71), and 33 (75%) were male. According to the Fitzpatrick scale, most patients had a fair (Type II) or medium (Type III) skin color (81%). In eight patients (18%), lower arm edema was present at the time of inclusion. Underlying etiologies included non-traumatic intracranial hemorrhage (41%), out-of-hospital cardiac arrest with poor neurologic prognosis (23%), and severe-traumatic brain injury (21%). Baseline characteristics did not differ between the training and test cohorts (Table 1).

**Table 1:** Baseline characteristics.

	All patients (n = 44)	Training 1 (n = 10)	Training 2 (n = 11)	Test (n = 23)
Male sex	33 (75%)	7 (70%)	9 (82%)	17 (74%)
Age (years)	65 (57–71)	58 (48–66)	68 (63–70)	65 (58–74)
BMI (kg/m^2^)	24.8 (23.4–27.7)	21.2 (19.9–24.3)	26.7 (24.7–29.4)	24.8 (23.5–27.7)
BSA (m^2^)	2.0 (1.8–2.1)	1.8 (1.6–2.1)	2.1 (2.0–2.2)	2.0 (1.9–2.1)
Medical history				
History of cardiac arrest	9 (21%)	2 (20%)	1 (9%)	6 (27%)
History of peripheral artery disease	2 (5%)	0	0	2 (10%)
History of atrial fibrillation	5 (12%)	1 (10%)	1 (9%)	3 (14%)
Cardiovascular risk factors				
Diabetes mellitus	4 (10%)	0	2 (18%)	2 (10%)
History of hypertension	18 (46%)	5 (50%)	7 (64%)	6 (33%)
Smoking, current or former	11 (61%)	3 (75%)	3 (50%)	5 (63%)
Underlying etiology				
Non-traumatic intracranial hemorrhage	18 (41%)	4 (40%)	5 (46%)	9 (39%)
Out-of-hospital cardiac arrest with poor prognosis	10 (23%)	2 (20%)	1 (9%)	7 (30%)
Infectious disease	2 (5%)	1 (10%)	1 (9%)	0
Severe-traumatic brain injury	9 (20%)	0	4 (36%)	5 (22%)
Overdose	2 (5%)	2 (20%)	0	0
Other^a^	3 (7%)	1 (10%)	0	2 (9%)
Fitzpatrick skin color scale				
I–White	6 (14%)	4 (40%)	1 (9%)	1 (4%)
II–Fair	23 (52%)	3 (30%)	6 (55%)	14 (61%)
III–Medium	12 (27%)	1 (10%)	4 (36%)	7 (30%)
IV–Olive	2 (5%)	2 (20%)	0	0
V–Brown	0	0	0	0
VI–Very dark brown	1 (2%)	0	0	1 (4%)
Arm hair density				
Nil	14 (32%)	5 (50%)	3 (27%)	6 (26%)
Sparse	11 (25%)	1 (10%)	3 (27%)	7 (30%)
Moderate	16 (36%)	3 (30%)	5 (46%)	8 (35%)
Dense	3 (7%)	1 (10%)	0	2 (9%)
Lower arm edema	8 (18%)	1 (10%)	4 (36%)	3 (13%)
Arterial line location				
Radial artery	37 (84%)	9 (90%)	9 (82%)	19 (83%)
Femoral artery	4 (9%)	0	1 (9%)	3 (13%)
Brachial artery	1 (2%)	0	1 (9%)	0
Location unknown	2 (5%)	1 (10%)	0	1 (4%)

History of cardiac arrest was known in 43 patients (98%), history of peripheral artery disease in 41 (93%), history of atrial fibrillation in 43 (98%), hypertension in 39 (89%), diabetes mellitus in 31 (93%), smoking in 18 (41%).

BMI, body mass index; BSA, body surface area.

a Other underlying etiologies include severe dermatological condition, autoimmune systemic disease, and other neurological disease.

### Cardiac arrest characteristics

**Fig. 2:**
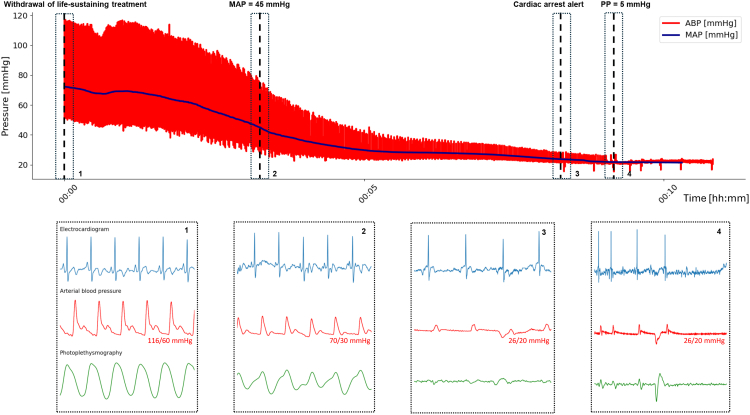
**Time course of the arterial blood pressure from withdrawal of life-sustaining treatment to death in a study participant.** The interval between withdrawal of life-sustaining treatment and death was approximately 10 min. The MAP fell below 45 mm Hg after 3.5 min, and the pulse pressure dropped below 5 mm Hg after 9 min. Four zoomed-in panels are shown below, displaying the ECG (blue), arterial blood pressure waveform (red), and photoplethysmography (green). MAP, mean arterial pressure; ABP, arterial blood pressure.

In all patients, cardiac arrest was based on a non-shockable cardiac rhythm (Fig. 2). The median length from time of withdrawal of life-sustaining treatment to cardiac arrest (pulse pressure <5 mmHg) was 21.5 min (IQR 13.5–79.5 min). Further cardiac arrest characteristics are presented in Table 2.

**Table 2:** Cardiac arrest characteristics.

	All patients (n = 44)	Training 1 (n = 10)	Training 2 (n = 11)	Test (n = 23)
Underlying rhythm				
Asystole	19 (44%)	3 (30%)	5 (50%)	11 (48%)
PEA	21 (49%)	7 (70%)	4 (40%)	10 (43%)
VF	0	0	0	0
VT	0	0	0	0
Unknown	3 (7%)	0	1 (10%)	2 (9%)
Arterial blood pressure data available at time of death	37 (84%)	10 (100%)	8 (73%)	19 (83%)
Baseline systolic blood pressure (mmHg)	115 ± 27	108 ± 29	116 ± 23	117 ± 28
Baseline diastolic blood pressure (mmHg)	59 (48–68)	50 (47–55)	52 (48–65)	62 (51–70)
Time from withdrawal of life-sustaining treatment to death, PP < 5 mmHg (min)	21.5 (13.5–79.5)	18 (10–30)	18 (16–60)	37.5 (15.5–152.5)
Hours of photoplethysmography data	331	65.5	93	172.5

Underlying rhythm was unknown in 3 cases (7%) (training 1; 0, training 2; 1, test; 2) due to missing ECG data at the time of death. In training cohort 2, one patient did not die during the monitoring period; this case was only assessed for false positive/clinically relevant alarms. Arterial blood pressure data at the time of death were available for 37 patients (84%). In Training 2, data were unavailable in three patients: one patient did not die during the monitoring period, and two patients had a malfunctioning arterial line near the time of death. In the test cohort, data were unavailable in four patients: three had no reference data available at all (see Fig. 1), and in one patient the reference data export terminated prematurely approximately 1 h before death.

PEA, pulseless electrical activity; VF, ventricular fibrillation; VT, ventricular tachycardia.

### Algorithm performance—training

The first training cohort consisted of ten patients and 65.5 h of photoplethysmography data. The sensitivity for the detection of cardiac arrest was 100% (95% CI 66–100%). Cardiac arrest was detected by the algorithm at a median MAP of 35 mmHg (IQR 25–41 mmHg) and a median pulse pressure of 18 mmHg (IQR 12–25 mmHg). The algorithm's alerts preceded cardiac arrest (pulse pressure <5 mmHg) by a median of 3 min (IQR 1–4 min). In addition, two alerts occurred during episodes of hemodynamic instability and were considered clinically relevant. One false positive cardiac arrest occurred in 65.5 h of photoplethysmography data (corresponding to 0.015 per hour), resulting in a positive predictive value of 91% (95% CI 57–100%).

Based on results from training cohort 1, the detection interval was extended from 10 to 20 s to reduce alerts for short-lasting episodes of hemodynamic instability. This resulted in a preserved sensitivity of 100% (95% CI 66–100%), a reduction from two clinically relevant alerts to one, and an unchanged false positive alert (n = 1) (see Supplement 2, Table S1).

The second training cohort consisted of 11 patients and 93 h of photoplethysmography data. One patient did not die during the monitoring period; this study participant was discharged from the ICU before circulatory arrest because of a prolonged time to death. Nine out of ten cardiac arrests were correctly recognized by the algorithm, resulting in a sensitivity of 90% (95% CI 54–99%). One arrest was detected outside the predefined time window (i.e. 3.5 min after pulse pressure <5 mmHg) and was therefore classified as false negative. Cardiac arrest was detected at a median MAP of 33 mmHg (IQR 22–47 mmHg) and a median pulse pressure of 7 mmHg (IQR 4–13 mmHg). The algorithm's alerts preceded cardiac arrest by a median of 3 min (IQR 1.5–4.5 min). In addition, four alerts occurred during episodes of hemodynamic instability and were considered clinically relevant. The median length of these alerts was 2 min. One false positive cardiac arrest alert occurred in 93 h of photoplethysmography data (corresponding to 0.011 per hour), resulting in a positive predictive value of 90% (95% CI 54–99%).

In training cohort 2, three cardiac arrest alerts were cancelled prematurely, followed by redetection of death within 10 s. Therefore, we increased the number of photoplethysmography peaks required for termination of the cardiac arrest alert from 4 to 10. This resulted in a sensitivity of 100% (95% CI 66–100%), a reduction from four clinically relevant alerts to one, and an unchanged false positive alert (n = 1) (see Supplement 2, Table S1).

### Algorithm performance—test

The test cohort consisted of 23 patients and 172.5 h of photoplethysmography data. The sensitivity for the detection of cardiac arrest was 100% (95% CI 82–100%). The algorithm detected the cardiac arrests at a median MAP of 30 mmHg (IQR 24–35 mmHg) and a median pulse pressure of 13 mmHg (IQR 11–19 mmHg). The algorithm's alerts preceded cardiac arrest by a median of 2.5 min (IQR 1–6 min). There was one false positive cardiac arrest alert in 172.5 h of photoplethysmography data (corresponding to 0.006 per hour), resulting in a positive predictive value of 96% (95% CI 77–100%). Table 3 presents the performance of the cardiac arrest detection algorithm in the training and test cohorts. The recall, specificity, precision, accuracy and F1-score are provided in the Supplement 2 (Table S2).

**Table 3:** Algorithm performance.

	Training 1 (n = 10)	Training 2 (n = 11)	Test (n = 23)
Sensitivity for cardiac arrest detection (95% CI)	100% (66–100%)	90% (54–99%)	100% (82–100%)
True positive cardiac arrest alerts	10	9	23
False positive cardiac arrest alerts	1	1	1
Clinically relevant alerts	2	4	0
Positive predictive value (95% CI)	91% (57–100%)	90% (54–99%)	96% (77–100%)
MAP at the time of cardiac arrest alert (mmHg)	35 (25–41)	33 (22–47)	30 (24–35)
Pulse pressure at the time of cardiac arrest alert (mmHg)	18 (12–25)	7 (4–13)	13 (11–19)
Time between cardiac arrest detection and PP < 5 mmHg (minutes)	3 (1–4)	3 (1.5–4.5)	2.5 (1–6)

The MAP was calculated as the time-averaged arterial pressure by integrating the continuous arterial pressure waveform.

CI, confidence interval; MAP, mean arterial pressure; PP, pulse pressure.

## Discussion

This study provides first clinical evidence of the performance of an automated cardiac arrest detection algorithm based on wrist-derived photoplethysmography in a non-induced cardiac arrest setting. The algorithm detected cardiac arrest after withdrawal of life-sustaining treatment with high sensitivity with only one false positive alert in the test cohort. Findings also extend previous validation in induced shockable cardiac arrest demonstrating that non-shockable cardiac arrest can also be detected with high sensitivity, providing the first formal evidence of adequate performance in this end-of-life setting.

The photoplethysmography-algorithm was originally developed in patients undergoing short-lasting induced circulatory standstill during transcatheter aortic valve implantation (TAVI).^16^ In this setting, circulatory arrest is induced by rapid ventricular pacing combined with aortic balloon inflation. Subsequently, the algorithm was externally validated in patients with induced shockable cardiac arrest.^17^ The sensitivity for cardiac arrest detection in the current study was comparable to that observed in the TAVI cohort (98%; DETECT-1a) and higher than in the induced shockable cardiac arrest group (92%; DETECT-1B). This difference can be explained by the presence of occasional residual cardiac output in the short-lasting induced VTs in the latter study, whereas the current study involves a progression toward sustained and complete loss of pulse. Whether these promising sensitivities translate to real-world settings with different cardiac arrest etiologies and less controlled conditions, remains to be determined. In addition, patients studied to date represent a relatively homogeneous population; further evaluation in more diverse populations, including individuals with different skin tones, is needed.

As photoplethysmography reflects blood flow rather than electrical activity, both shockable and non-shockable arrests result in flattening of the photoplethysmography curve and were expected to be detected. However, whereas shockable arrests typically occur suddenly, non-shockable arrests may be preceded by progressive hypotension, depending on the underlying cause. Detection of cardiac arrest irrespective of the underlying rhythm is important, as 60–80% of all out-of-hospital cardiac arrests are non-shockable.^5^^,^^26^^,^^27^ Despite this, to date all wearable-based automated cardiac arrest detection research has focused on sudden cardiac arrest, which is easier to simulate or induce.^10^, ^11^, ^12^ Although this focus is understandable as automated detection of ventricular fibrillation cardiac arrest offers a high potential for survival, it has left the much more common non-shockable arrests unaddressed. Specifically, the ‘loss of pulse’ detection model assumes a rapid transition from a pulsatile to pulseless state.^13^ We have now shown that the algorithm in this study can also detect cardiac arrest following gradual hemodynamic decline.

As this was an initial evaluation in non-induced cardiac arrest, the rule-based model was optimized through two iterations prior to validation. Extending the detection interval to 20 s and increasing the required number of detected peaks to cancel an alarm reduced alarms triggered by short-lasting hemodynamic instability as well as repetitive alarms for the same episode. This revised configuration more closely reflects the intended future use of wearable-based out-of-hospital cardiac arrest detection, where alert relevance and the need to minimize false alarms favor longer detection windows.

All cardiac arrest alarms occurred shortly before the time of death in a context of withdrawal of life-sustaining treatment, where circulatory decline typically involves prolonged low-perfusion patterns.^19^ While the optimal timing of an alert is clear in cases of sudden cardiac arrest, it is less straightforward when cardiac arrest is preceded by progressive hemodynamic and respiratory deterioration, as is observed after withdrawal of life-sustaining treatment.^19^ In this context, alerts were classified as true positive if they occurred after withdrawal of life-sustaining treatment and within a time window of 15 min before to 2 min after the pulse pressure fell below 5 mmHg. At the time of the alerts, the median MAP was approximately 30–35 mmHg, indicating severe circulatory compromise requiring cardiopulmonary resuscitation.^28^ The post-arrest allowance accounted for the reported median 3-min interval between arrest onset and dispatcher alarm,^2^ and for the approximately 1-min processing time required for automated detection algorithms. Currently, the technology is primarily being developed to reliably detect cardiac arrest and subsequently trigger an alert to enable rapid assistance, rather than provide early warning in hemodynamic deterioration. Future research should investigate whether wrist-derived photoplethysmography signals can also identify pre-arrest patterns or hemodynamic instability, and whether such early warning would be clinically meaningful in home and hospital settings.

The three false positive alerts (durations of 57, 120 and 139 s, respectively) were not associated with arrhythmias or transient hemodynamic instability. In two of the three false alerts, the baseline amplitude of the green photoplethysmography signal was relatively low (a power of ∼50), which may have affected pulse detection by the algorithm. The red and infrared photoplethysmography signals during these episodes showed preserved waveform activity, warranting further investigation of a multi-wavelength approach. False positives should ideally be evaluated using recordings obtained during daily life which will be done in the ongoing DETECT-3 study.^8^ As data in the present study were collected in a hospital setting under relatively controlled conditions, the observed false positive rate may not reflect that encountered during real-world use, where motion artefacts and other signal disturbances occur more frequently. The impact of such artefacts may be mitigated by extending detection intervals and incorporating multi-wavelength and accelerometer data into the algorithm. In addition, false positive alerts may be reduced by allowing users to cancel alerts before emergency services are notified.^13^^,^^29^ These potential false alert mitigation measures were not appropriate in the present study, as patients were sedated and immobile throughout monitoring. False positive alerts are important to address, as they may place a significant burden on EMS and may contribute to alarm fatigue, potentially undermining the acceptability of the technology. If performance remains adequate under real-world conditions, subsequent studies should evaluate the effective integration of wearable-based cardiac arrest alerts into the chain of survival, including practical challenges such as victim localization and reachability, as well as the potential impact of wearable-based cardiac arrest detection on time to CPR initiation, survival and neurological outcomes.

Limitations of this study include the retrospective evaluation of algorithm performance and the relatively controlled study setting. The sample size was relatively small but based on a predefined sample size calculation and was sufficient to address the primary study objective. Furthermore, due to the limited sample size, algorithm modifications were evaluated on the same dataset used for optimization. Invasive arterial blood pressure data were unavailable at the time of death in a limited number of patients because of catheter malfunction. Although an invasive blood pressure reference standard was used, potential effect of arterial stenosis on blood pressure measurements was not assessed. Finally, data were collected exclusively in sedated patients, precluding assessment of algorithm performance during movement. Prospective validation in real-world settings is therefore required.

In conclusion, cardiac arrest following withdrawal of life-sustaining treatment can be accurately detected using a wrist-derived photoplethysmography-algorithm. These findings provide the first formal evidence in automatically detecting cardiac arrest events outside the induced setting, supporting further development of this potentially lifesaving technology. As a next step, validation in daily-life settings and across diverse populations is required to assess sensitivity and false positives in real-world use. If proven effective in these settings, wearable-based cardiac arrest detection could in the future enable earlier recognition of out-of-hospital cardiac arrest.

## Contributors

RE: Conceptualization, Data Curation, Formal Analysis, Investigation, Methodology, Project Administration, Visualization, Writing–Original Draft.

CJ: Data Curation, Formal Analysis, Investigation, Methodology, Writing—Review & Editing.

LP: Data Curation, Formal Analysis, Investigation, Methodology, Writing—Review & Editing.

KE: Data Curation, Formal Analysis, Investigation, Methodology, Project Administration, Resources, Software, Writing—Review & Editing.

RvK: Conceptualization, Data Curation, Methodology, Resources, Software, Validation, Writing—Review & Editing.

ER: Conceptualization, Funding Acquisition, Supervision, Writing—Review & Editing.

MB: Conceptualization, Funding Acquisition, Methodology, Supervision, Writing—Review & Editing.

RB: Conceptualization, Funding Acquisition, Methodology, Supervision, Writing—Review & Editing.

AC: Methodology, Formal Analysis, Resources, Supervision, Writing—Review & Editing.

PS: Conceptualization, Funding Acquisition, Project Administration, Resources, Software, Writing—Review & Editing.

EB: Conceptualization, Funding Acquisition, Supervision, Writing—Review & Editing.

CH: Conceptualization, Methodology, Software, Supervision, Validation, Writing—Review & Editing.

NvR: Conceptualization, Funding Acquisition, Project Administration, Resources, Supervision, Writing—Review & Editing.

JB: Conceptualization, Formal Analysis, Funding Acquisition, Methodology, Project Administration, Resources, Validation, Writing–Original Draft, Writing—Review & Editing.

Authors that have accessed and verified the data: RE, CJ, LP, KE, JB. Authors responsible for the decision to submit the manuscript: RE, JB.

## Data sharing statement

No individual participant data will be made available. Participating patients and/or their relatives were not asked to provide consent for reuse of data in other studies. Due to the sensitive nature of the setting and agreements with the medical ethics committee, data from the DETECT-1C study cannot be shared.

## Declaration of interests

RE received a research grant from the European Resuscitation Council not related to this manuscript; is editorial board member of Resuscitation Plus. NvR received a research grant from the Dutch Heart Foundation and Radboud University Medical Center related to this manuscript; received research grants from Biotronik, Abbott, Medtronic, and Philips, not related to this manuscript; received speaker fees from Abbott and Bayer. JB received a research grant from ZonMw not related to this manuscript. All other authors declare no competing interests.

## Appendix A Supplementary data

Supplement Revised
