# Supplementary material for: Automated cardiac arrest detection using wrist-derived photoplethysmography during withdrawal of life-sustaining treatment: a prospective clinical validation study
Source: Lancet Reg Health Eur. 2026 Jul 24;67:101791. doi: 10.1016/j.lanepe.2026.101791 (PMC13427576; doi:10.1016/j.lanepe.2026.101791)
Supplement: Supplement Revised [file mmc1.docx]

**Supplement 1 – Patient selection and informed consent**

**Patient selection**

Patients admitted to the adult Intensive Care Unit (ICU) in whom withdrawal of life-sustaining treatment was planned due to poor prognosis were eligible for inclusion, irrespective of respiratory support. There were no specific criteria regarding age, length of hospital stay, or underlying etiology. The assessment of a poor prognosis was made exclusively by the attending intensivist, and the research team was not involved in this decision in any way.

Inclusion criteria:

- Planned withdrawal of life-sustaining treatment at the intensive care unit
- Presence of an arterial line inserted for invasive blood pressure monitoring
- Age ≥ 18 years
- Fitting the wristband

Exclusion criteria:

- Known bilateral hemodynamically relevant subclavian artery stenosis
- Medical issues interfering with wearing of the wristband (e.g. skin disorders or bandaging)
- Ongoing mean arterial pressure despite vasopressors use of <60 mmHg prior to inclusion
- Unavailability of the wristband used for photoplethysmography recording
- Patient is scheduled for organ donation as a heart-beating donor

**Informed consent procedure**

A researcher contacted the ICU daily to identify eligible patients. Screening was performed by ICU staff. If a patient was deemed eligible, the attending ICU physician assessed whether it was appropriate to approach the patient or relatives regarding study participation. If considered appropriate, the ICU physician or nurse asked whether the patient or relatives were open to receiving study information from the researcher. Only with their permission was study information provided by the researcher. In cases where relatives indicated they did not feel up to receiving study information, no further approach by the researcher took place and the patient was not enrolled.

In total, 48 study information conversations took place, and verbal informed consent was obtained in 45 cases. Reasons for non-participation included unwillingness to participate or the presence of bandaging on both forearms precluding wristband placement. In accordance with the Medical Ethics Committee East-Netherlands, verbal informed consent was considered sufficient for participation. Consent was documented in the electronic patient file.

**Supplement 2 – Supplementary tables**

**Table S1. Effect of model adjustments on algorithm performance.**

|  | **Training 1**  Not adjusted | **Training 1**  Adjusted | **Training 2**  Not adjusted | **Training 2**  Adjusted | **Test** |
| --- | --- | --- | --- | --- | --- |
| **Settings of the cardiac arrest detection model** | | |  |  |  |
| Detection window (s) | 10 | 20 | 20 | 20 | 20 |
| Number of photoplethysmography peaks for redetection | 4 | 4 | 4 | 10 | 10 |
| **Output of the cardiac arrest detection model** | | |  |  |  |
| Total number of alerts | 13 | 12 | 15 | 12 | 23 |
| True positive | 10 | 10 | 9 | 10 | 23 |
| False positive | 1 | 1 | 1 | 1 | 1 |
| Clinically relevant alert | 2 | 1 | 4 | 1 | 0 |

Based on the results from Training 1, the detection window was prolonged from 10 to 20 seconds. Based on the results from Training 2, the number of photoplethysmography peaks required for termination of a cardiac arrest alert was increased from 4 to 10. Alerts were considered clinically relevant in case of hemodynamic instability (MAP ≤45 mmHg or pulse pressure ≤15 mmHg).

**Table S2. Algorithm performance expressed as precision, recall, F1-scores, accuracy, and specificity with 95% confidence intervals (CI).**

|  | **Training 1**  (n=10) | **Training 2**  (n=11) | **Test**  (n=23) |
| --- | --- | --- | --- |
| Recall (95% CI) | 1.00 (0.66-1.00) | 0.90 (0.54-0.99) | 1.00 (0.82-1.00) |
| Precision (95% CI) | 0.91 (0.57-1.00) | 0.90 (0.54-0.99) | 0.96 (0.77-1.00) |
| F-1 score (95% CI) | 0.95 (0.61-1.00) | 0.90 (0.54-0.99) | 0.98 (0.79-1.00) |
| Accuracy (95% CI) | 99.8% (99.6-99.9%) | 99.9% (99.7-100%) | 100% (99.9-100%) |
| Specificity (95% CI) | 99.7% (99.5-99.8%) | 99.9% (99.6-100%) | 100% (99.9-100%) |

Recall was defined as the proportion of correctly identified cardiac arrests divided by the total number of cardiac arrests. Precision was defined as the proportion of correctly identified cardiac arrests divided by all positive alerts (true positives and false positives). F1-score was calculated as the harmonic mean of recall and precision. Accuracy was calculated as the proportion of detection windows correctly classified as either cardiac‑arrest (true positives) or non-cardiac arrest (true negatives), divided by the total number of detection windows. Specificity was calculated as the proportion of detection windows containing pulsatile photoplethysmography activity that were correctly classified as non–cardiac‑arrest (true negatives).
